# HArmonyCa™ hybrid filler to restore connective tissue: An Italian real‐life retrospective study

**DOI:** 10.1111/jocd.16503

**Published:** 2024-10-03

**Authors:** Ilaria Proietti, Alessandra Spagnoli, Alison Favaroni, Alessandro Gritti, Marco Dal Canton, Sandro Quartucci, Chantal Sciuto, Dario Bertossi, Mariagrazia Patalano, Maurizio Cavallini, Maria Teresa Saliani, Nicola Kefalas, Enrica Angelone, Selene Mogavero

**Affiliations:** ^1^ Department of Medical‐Surgical Sciences and Biotechnologies, Polo Pontino Sapienza University of Rome Rome Italy; ^2^ Department of Public Health and Infectious Diseases Sapienza University of Rome Rome Italy; ^3^ Scientific Writing Torgiano (PG) Italy; ^4^ Chirurgia Maxillo Facciale Milan Italy; ^5^ Ambulatory of Medical and Surgical Dermatology Belluno Italy; ^6^ Private Practice 1 Rome Italy; ^7^ Private Practice 2 Rome Italy; ^8^ Section of Oral and Maxillofacial Surgery, Department of Surgical Sciences University of Verona Verona Italy; ^9^ Private Practice Messina Italy; ^10^ Department of Operative Unit Dermatology and Dermatosurgery Milan Italy; ^11^ Private Practice Modugno (BA) Italy; ^12^ Private Practice Torino Italy; ^13^ Private Practice Avezzano (AG) Italy

**Keywords:** calcium hydroxylapatite, face rejuvenation, HArmonyCa™, hyaluronic acid, hybrid filler

## Abstract

**Background:**

Facial aging and dermal conditions may negatively influence the quality of life, leading patients to seek aesthetic procedures to restore a more satisfying appearance. HArmonyCa™ is a recently developed hybrid filler that combines the actions of the most common dermal fillers, hyaluronic acid (HA) and calcium hydroxylapatite (CaHA).

**Aims:**

This study investigates the efficacy and safety of HArmonyCa™ in patients affected by chrono‐ and photoaging and several facial skin conditions.

**Patients/Methods:**

One hundred and twenty‐nine patients, affected by chrono‐ and photoaging, and skin conditions such as oily and acne‐prone skin, rosacea, or scarring, were treated with HArmonyCa™. Injections followed the retrograde linear fanning technique. A physicians' consensus identified five optimal entry points. The physician and patients assessed treatment outcomes using the Global Aesthetic Improvement Scale (GAIS) 9 months after treatment (including immediate lift effect, skin firmness, and elasticity), and 3D images were taken for documentation. Adverse events (AEs) were evaluated immediately after the procedure and after 9 months.

**Results:**

According to the physician's assessments, all patients displayed an improvement in facial appearance, particularly during movement, with the patients' evaluation showing agreement. Only minor AEs were reporte, which resolved spontaneously. Moreover, HArmonyCa™ treatment proved compatible with different medications and aesthetic procedures.

**Conclusions:**

This study shows that one treatment with HArmonyCa™ yields highly satisfactory outcomes in patients affected by skin conditions. For the first time, we show that HArmonyCa™ is a dynamic filler that improves facial laxity during movement. The treatment proved to be safe and fully compatible with other cosmetic procedures and medications.

## 1 INTRODUCTION

Facial skin stands out as the most prominently visible organ of the body. Its state can deteriorate due to several factors, including aging and dermal conditions.^1^ Aging processes are influenced by intrinsic factors (chronological aging) and extrinsic factors, such as pollution, smoking, and UV irradiation (photoaging).^2^ It is estimated that photoaging is responsible for 80% of premature facial aging.^1^ Interestingly, both intrinsic and extrinsic aging can be traced to the same molecular pathways, leading to a decreased amount and organization of collagen and elastic fibers, skin laxity, and reduction in hyaluronic acid (HA).^3^, ^4^, ^5^ Acne and rosacea rank among the most prevalent conditions impacting facial skin, leading to scarring, skin hyperpigmentation, and erythema.^6^

Facial and skin changes due to intrinsic‐ and photo‐aging and different dermal conditions can negatively influence the patient's quality of life, generating discomfort in social interactions and the perception of self‐beauty. Cosmetic and rejuvenation procedures are therefore highly requested.^7^ HA‐based fillers are the most widely used to restore the lost volume of the face and its contour.^8^, ^9^ Yet, HA fillers lack the ability to improve the thickness and the texture of the dermis. Moreover, HA is naturally reabsorbed as a component of the dermis, making its action temporary, lasting 6–12 months.^8^, ^10^

To implement HA action on facial rejuvenation, HA fillers have recently been used in combination with calcium hydroxylapatite (CaHA)‐based fillers either as separate injections during the same procedure or as a freshly mixed formulation before the procedure (off‐label).^11^ CaHA is the second most used injectable filler and consists of microspheres containing calcium and phosphate. As these minerals are components of the bones, CaHA fillers are biocompatible.^9^ CaHA microspheres have a biostimulatory effect, inducing fibroblast intervention and leading to neocollagenesis. The collagen formation induced by CaHA contributes to the volumization effect and provides a lifting effect, improving skin texture and laxity. Moreover, due to its characteristics, CaHA has longer‐lasting results than HA fillers.^12^, ^13^ Recently, a new hybrid filler has been developed: HArmonyCa™ is a ready‐to‐use formulation composed of HA and CaHA microspheres, combining the advantages of both fillers in one syringe.^4^ Being HArmonyCa™ a recently developed filler, only a few studies have been performed so far on it; it has, however, been shown that patients receiving this filler achieved facial augmentation, lifting effects, and improved skin laxity after a single procedure, with results lasting for at least 1 year.^4^, ^5^, ^13^, ^14^ HArmonyCa™ has also been reported to be beneficial in patients affected by skin conditions such as actinic elastosis and facial lipoatrophy.^3^, ^15^

In this study, we analyzed the efficacy and safety of the hybrid HA/CaHA filler (HArmonyCa™) on aesthetic facial rejuvenation of 129 patients also affected by different skin conditions. We then evaluated the compatibility of this procedure with other factors. Moreover, we assessed the patients' satisfaction levels over a 9‐month follow‐up period. 3D images were collected to show the patients in different facial positions and during the pinch test. This allowed to investigate, for the first time, the properties of HArmonyCa™ as a dynamic filler, improving the mechanical properties of the face during movement.

## 2 MATERIALS AND METHODS

### 2.1 Patients' selection

This retrospective, real‐life study was performed at our dermatological clinic in Italy, between April 2021 and January 2023. The aim of the study was to investigate the efficacy, safety, compatibility with other factors, and level of satisfaction in patients who underwent an aesthetic facial rejuvenation procedure of soft tissue augmentation with the hybrid HA/CaHA filler (HArmonyCa™). Patients were also affected by facial skin conditions. The study included 129 patients (110 females and 19 males) above 18 years old. The skin type of each patient was categorized according to the Fitzpatrick Skin Phototype Classification^16^ and ranged between categories I and IV with the following definitions: “light, pale white” (I), “white, fair” (II), “medium white to olive” (III) and “olive tone” (IV). Patients in this study presented one or more skin condition(s), including chrono‐ and photoaging, oily and acne‐prone skin, rosacea, and scarring. Before treatment, a clinical evaluation was performed for each patient to investigate the presence of systemic diseases, pharmacological therapies, and other cosmetic procedures (Table 1). This study respected the ethical guidelines of the Declaration of Helsinki, ensuring every participant's safety, well‐being, and rights (Ethical Committee protocol n. 0097231/2023). All patients gave informed consent after an exhaustive explanation of the procedure and its potential adverse events (AEs).

**TABLE 1:** Patients' baseline characteristics.

Total patients	129 (100)
Sex	
Female	110 (85.3)
Male	19 (14.7)
Age	
(18–40)	9 (7.0)
(41–50)	30 (23.3)
(51–60)	58 (45.0)
(>60)	32 (24.8)
Fitzpatrick	
I	3 (2.3)
II	42 (32.6)
III	66 (51.2)
IV	18 (14.0)
Facial skin conditions	
OAPS	20 (15.5)
OAPS, photoaging	1 (0.8)
OAPS, rosacea, scarring	1 (0.8)
Photoaging	68 (52.7)
Photoaging, scarring	1 (0.8)
Rosacea	9 (7.0)
Rosacea, scarring	1 (0.8)
Scarring	28 (21.7)
Previous treatments	
Botulinum toxin injections	20 (15.5)
Other filler injections	18 (14.0)
Laser	41 (31.8)
Surgery	7 (5.4)
None	43 (33.3)
Systemic diseases	
Cardiovascular	11 (8.5)
Infection	10 (7.8)
Oncologic	5 (3.9)
Autoimmune	1 (0.8)
None	102 (79.1)
Use of medications	
Anticoagulants	11 (8.5)
Antibiotics, antivirals, antimycotics	10 (7.8)
Chemotherapeutics	5 (3.9)
Immunosuppressants	1 (0.8)
None	102 (79.1)

*Note*: Values are given as *n* (%).

Abbreviation: OAPS, oily and acne‐prone skin.

### 2.2 Image acquisition process

The present study employed the Vectra H1 imaging system (Canfield Scientific, NJ, USA) for the acquisition of pre‐ (T_0_) and post‐treatment images 9 months after the infiltration of HArmonyCa™ (T_1_). This imaging system is composed of compact 3D cameras that rely on the principle of passive stereophotogrammetry. Three images are acquired from different perspectives and are then overlayed into a single 3D image. The cameras include a flash system and a dual‐beam pointer to standardize the photographing distance. The Vectra H1 system, chosen for its capacity to deliver high‐resolution, standardized imagery, was utilized to meticulously document facial features at rest, during a smile, through pinch tests, and in support along the mandibular line. Rigorous positioning protocols were followed before image capture to ensure consistency and facilitate accurate comparison between pre‐ and post‐treatment conditions. This methodological precision, coupled with the advanced capabilities of Vectra H1, enabled a comprehensive and detailed analysis of the long‐term effects of HArmonyCa™ infiltration on facial aesthetics, particularly in varying expressions and along the mandibular line. The resulting imagery serves as a valuable resource for objectively evaluating treatment outcomes, contributing to the scientific understanding of the impact of HArmonyCa™ on facial harmony and support.

### 2.3 Technical procedures

The hybrid filler HArmonyCa™ was employed for the aesthetic treatment of patients with skin conditions. HArmonyCa™ (Allergan Aesthetics, AbbVie Company) is a new injectable filler, composed of 20 mg/mL HA, 55.7% CaHA microspheres 25–45 μM and 0.3% lidocaine, contained in a 1.25 mL syringe. The presence of the anesthetic lidocaine is essential to reduce the discomfort of the procedure. The use of HArmonyCa™ has already been approved in several countries.^5^

In preparation for the procedure, skin antiseptic treatment of the targeted facial areas was performed using 0.5% alcoholic chlorhexidine. Then, 30 min before the procedure, an anesthetic cream containing 5% lidocaine was applied to the targeted regions and entirely removed immediately before the treatment. The patients were positioned supine at a 45° angle to optimize their comfort.

**FIGURE 1:**
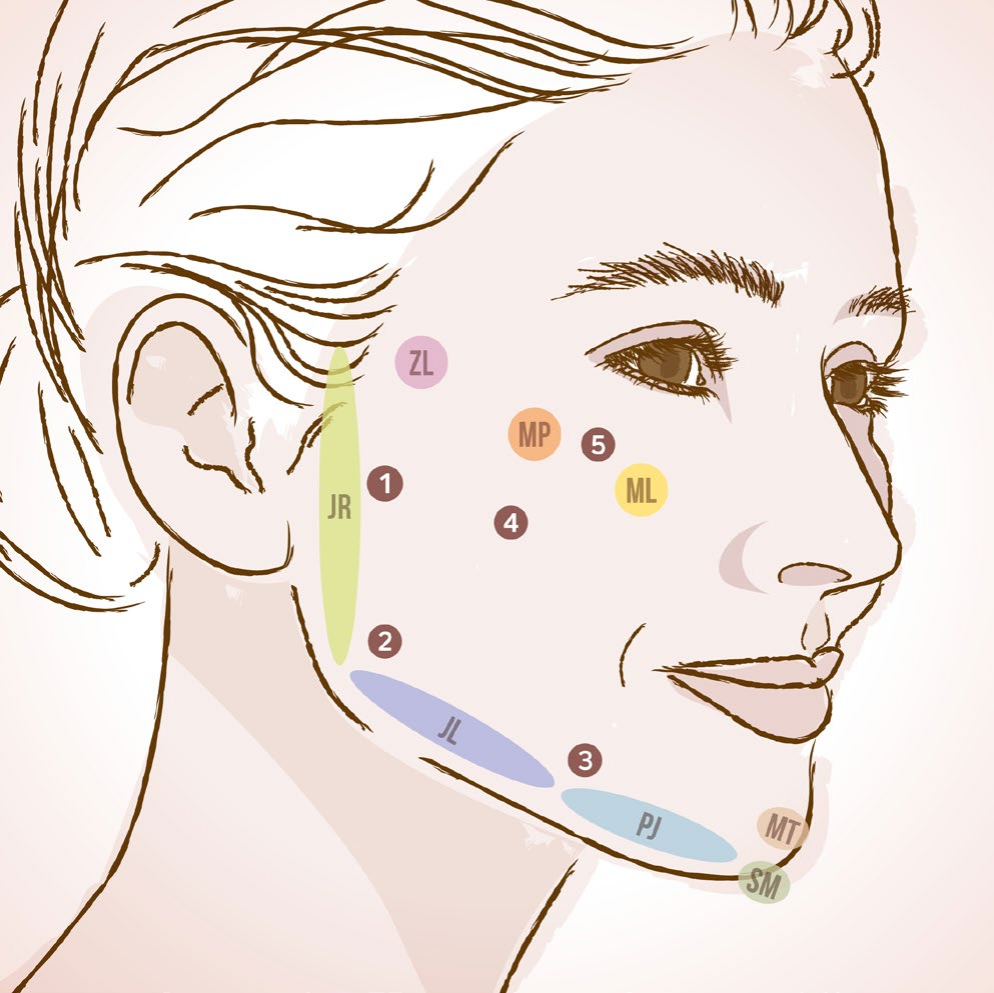
Anatomical areas of treatment (ML, malar lateral; MP, malar prominence; ZL, zygoma lateral; MT, mental; SM, submental; PJ, prejowl; JL, jawline; JR, jaw ramus),^17^ and entry points for the injection of HArmonyCa™ (1, the subzygomatic region; 2, the region over the mandibular body, at the anterior edge of the masseter muscle; 3, the prejowl sulcus; 4, the parotid area; 5, the zygomatic or malar area).

Different areas of the face's middle third, lower third, and lateral portion were targeted (Figure 1).^14^, ^17^ Injections of HArmonyCa™ were performed through skin perforations at different entry points. To reach all the targeted areas, a consensus among physicians was reached, identifying five optimal entry points to inject and deliver HArmonyCa™: the subzygomatic region, the region over the mandibular body, at the anterior edge of the masseter muscle, the prejowl sulcus, the parotid area, and the zygomatic, or malar area (Figure 1). The product was placed in the superficial subcutaneous layer, using a 22‐gauge cannula and following a retrograde linear fanning technique.^14^ The perforated skin areas were gently massaged and compressed. According to the number of treated areas and to the physician's evaluation of the face at baseline, between one and four syringes of HArmonyCa™ were administered (1.25–5 mL), with a range of 0.625–2.5 mL product for hemiface.

### 2.4 Data evaluation

All patients received a follow‐up visit 9 months after the procedure. Physicians and patients evaluated the procedure outcomes independently by assessing the improvements in face quality at the follow‐up visit, compared to the face condition at baseline. In addition, physicians assessed the occurrence of AEs immediately after the procedure and at the 9‐month follow‐up visit. AEs were classified as immediate AEs if they occurred within 1 month of the procedure, and as late AEs if they occurred after more than 1 month of the procedure. The evaluations were conducted both live and on the images provided by the Vectra H1 imaging system. An adapted Global Aesthetic Improvement Scale (GAIS) was used,^18^ employing the definitions “much improved,” “improved,” “no change,” and “worse.” This adapted GAIS considered several outcomes, such as skin consistency, immediate lift effect, skin firmness, and elasticity.

### 2.5 Statistical analysis

All statistical analyses were performed using R software (version 4.0.2). Comparisons between groups were conducted using the Chi‐squared test. The Cohen's kappa index was applied to assess the agreement between the improvements in the GAIS perceived by the physician and the patients. All *p*‐values were two‐sided, and *p* < 0.05 was considered as significant.

## 3 RESULTS

### 3.1 Study population at baseline

This study includes 129 patients (110 females and 19 males), the majority (69.8%) over 50 years old. The patients' skin types refer to the Fitzpatrick Skin Phototype Classification^16^ and belong to categories I to IV. Most of the patients were assigned to categories II and III, with 66 patients in category III (medium white to olive) and 42 patients in category II (white, fair) (Table 1). At baseline, all patients presented one or more skin condition(s). Photoaging was the most prevalent, with 68 patients (52.7%) affected. Other patients presented oily and acne‐prone skin, rosacea, scarring, or several conditions simultaneously (Table 1). In the 6 months prior to the HArmonyCa™ treatment, 86 patients (66.7%) underwent a different cosmetic procedure, such as laser, injections of botulinum toxin, or other filler, and surgery (Table 1). At baseline, 27 patients (20.9%) were affected by a systemic disease and received medication for it (Table 1).

### 3.2 Treatment outcomes

Patients were treated with HArmonyCa™ in different areas of the face, as described in Table 2 (Figure 1). Interestingly, while different areas combinations were treated, the jaw ramus (JR) was treated in all 129 patients. Nine months after the treatment, the outcomes were evaluated. According to the physicians' GAIS evaluation, 100% of patients showed improvement in the appearance of the face and skin quality, with the majority of patients (61.2%) evaluated as “much improved” (Table 2). When considering the broader categories of skin conditions, as highlighted in Table 3, patients with “UV‐related” conditions (photoaging and rosacea) displayed a higher frequency of “much improved” evaluated outcomes compared to patients affected by “general scarring” conditions (*p* = 0.001, Chi‐squared test) (Table 3).

**TABLE 2:** Treatment outcomes and patients' characteristics at the nine‐month follow‐up visit.

Treated areas	
JL, JR, MT	1 (0.8)
JL, JR, PJ	24 (18.6)
JL, JR, MT, PJ, SM	19 (14.7)
JL, JR, ZL	31 (24.0)
JR, ML, MP, ZL	54 (41.9)
GAIS assessed by physician	
Much improved	79 (61.2)
Improved	50 (38.8)
GAIS assessed by patient	
Much improved	81 (62.8)
Improved	45 (34.9)
No change	2 (1.6)
Worse	1 (0.8)
Immediate AEs	
Bruises	5 (3.9)
Erythema	94 (72.9)
Erythema, swelling	1 (0.8)
Infection	1 (0.8)
Swelling	14 (10.9)
None	14 (10.9)
Late AEs	
Nodules	1 (0.8)
Swelling	5 (3.9)
None	123 (95.3)
Subsequent treatments	
Botulinum toxin injections	36 (27.9)
Other filler injections	20 (15.5)
Laser	18 (14.0)
Surgery	2 (1.6)
None	53 (41.1)
Loss of weight	
<5 Kg	91 (70.5)
>5 Kg	32 (24.8)
NA	6 (4.7)

*Note*: Values are given as *n* (%).

Abbreviations: AE, adverse event; GAIS, Global Aesthetic Improvement Scale; JL, jawline; JR, jaw ramus; ML, malar lateral; MP, malar prominence; MT, mental; PJ, prejowl; SM, submental; ZL, zygoma lateral.

**TABLE 3:** Association between facial skin conditions and GAIS perceived by the physician.

	Physicians' GAIS	
	Much improved	Improved
Facial skin conditions—*n* (%)		
General scarring	21 (42.0)	29 (58.0)
OAPS	10 (50.0)	10 (50.0)
OAPS, photoaging	0 (0.0)	1 (100.0)
OAPS, rosacea, scarring	1 (100.0)	0 (0.0)
Scarring	10 (35.7)	18 (64.3)
UV‐related skin conditions	58 (73.4)	21 (26.6)
Photoaging	49 (72.1)	19 (27.9)
Photoaging, scarring	1 (100)	0 (0.0)
Rosacea	7 (77.8)	2 (22.2)
Rosacea, scarring	1 (100)	0 (0.0)

*Note*: The chi‐squared test performed on the general facial skin condition groups (“general scarring” and “UV‐related skin conditions”) obtained a *p*‐value of 0.001.

Abbreviations: GAIS, Global Aesthetic Improvement Scale; OAPS, oily and acne‐prone skin.

**FIGURE 2:**
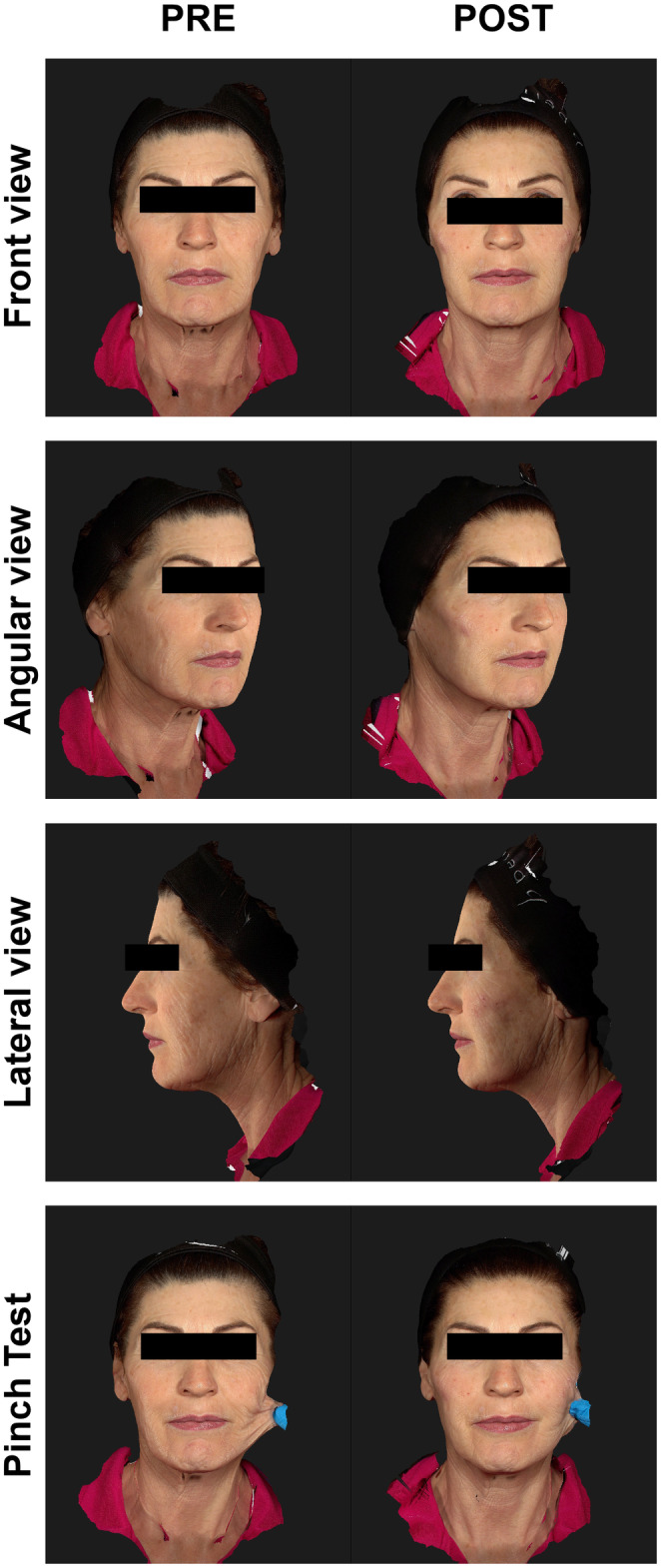
This 48‐year‐old woman had severe photoaging and skin laxity. She was treated with two vials of HArmonyCa™. Images are taken at rest, with the last image at the bottom showing the “pinch test.” PRE, images taken before the procedure; POST, images taken at the 9‐month follow‐up.

**FIGURE 3:**
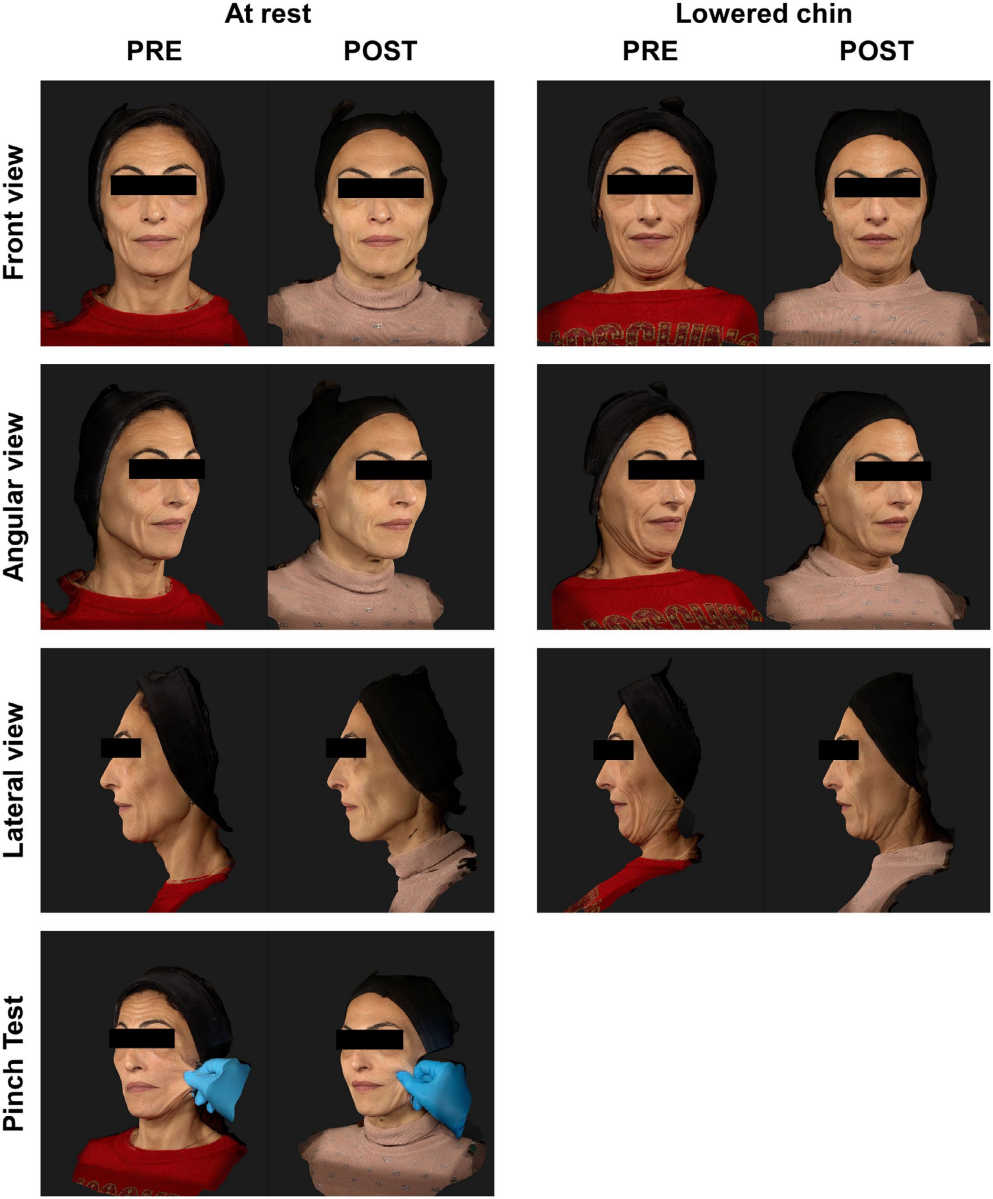
This 49‐year‐old woman had severe skin laxity and chronoaging. She was treated with two vials of HArmonyCa™ in the lower third and also had a botulinum toxin injection in the forehead. On the left are images taken at rest, with the last image at the bottom showing the “pinch test.” On the right are images taken while the patient was lowering her chin. PRE, images taken before the procedure; POST, images taken at the 9‐month follow‐up.

**FIGURE 4:**
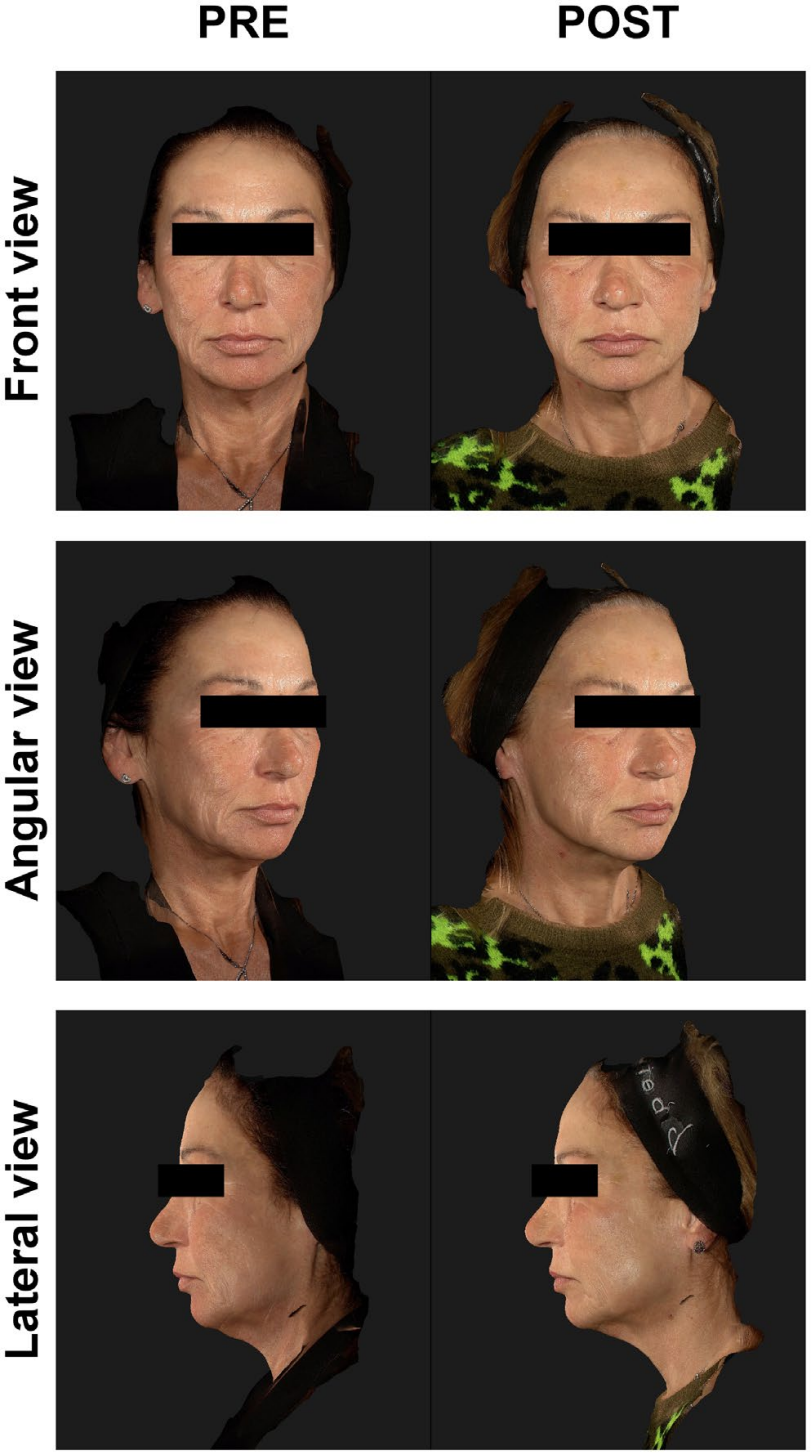
This 56‐year‐old woman had post‐acne scarring and photoaging. She was treated with four vials of HArmonyCa™. Images are taken at rest. PRE, images taken before the procedure; POST, images taken at the 9‐month follow‐up.

Interestingly, one single treatment with HArmonyCa™ significantly enhances the skin's elasticity, reducing laxity. These improvements are significantly visible during the pinch test and movement, such as smiling and lowering the chin (Figures 2 and 3). However, changes in skin quality are also evident in a rest state (Figure 4).

In this study, the patients were also asked to independently assess the outcome of their treatment following the GAIS. According to the patients' evaluations, 97.7% of the treatments have been successful. In only two cases the GAIS assessed by the patients did not find any change compared to the baseline and in one case the result was judged as “worse” (Table 2). Nonetheless, the GAIS assessed by the patients were in line with the physicians' evaluations, showing significant concordance between physician‐perceived and patient‐perceived GAIS improvement (Cohen's concordance index *k* = 0.435, *p* < 0.001) (Table 4).

**TABLE 4:** Agreement between improvement perceived by the physician and the patient.

	Physicians' GAIS—*n* (%)	
	Much improved	Improved
Patients' GAIS		
Much improved	64 (81.0)	17 (34.0)
Improved	15 (19.0)	30 (60.0)
No change	0 (0.0)	2 (4.0)
Worse	0 (0.0)	1 (2.0)

Abbreviation: GAIS, Global Aesthetic Improvement Scale.

In general, treating different facial areas with HArmonyCa™ proved to be an efficient approach in patients with different skin conditions. Moreover, patients and physicians' evaluations displayed great agreement, with patients showing high satisfaction.

### 3.3 Adverse events

To evaluate the safety of HArmonyCa™ injections in the presence of skin conditions, AEs were assessed by the physicians immediately after the procedure (immediate AEs) and during the nine‐month follow‐up visit (late AEs). The immediate reactions can be associated with the technical procedure, with erythema and swelling being the most common. Fourteen patients did not show any sign of immediate AEs. At the 9‐month follow‐up, only six patients presented minor reactions, while most patients (95.3%) showed no sign of AEs (Table 2). All AEs registered in this study can be categorized as minor and healed spontaneously. Of all patients who reported immediate AEs, the majority did not report any AE at the 9‐month follow‐up visit. In particular, of the 94 patients who reported erythema as immediate AE, only five reported the presence of nodules or swelling after 9 months, suggesting that these are sensitive patients with intrinsic reactivity. However, the late AEs were also minor and resolved spontaneously without medical intervention (Table 5). Altogether, these data indicate that HArmonyCa™ injections are safe.

**TABLE 5:** Association between immediate and late adverse events.

	Late AEs—*n* (%)		
	Nodules	Swelling	None
Immediate AEs—*n* (%)			
Bruises	0 (0.0)	0 (0.0)	5 (100.0)
Erythema	1 (1.1)	4 (4.3)	89 (94.7)
Erythema, swelling	0 (0.0)	0 (0.0)	1 (100.0)
Infection	0 (0.0)	0 (0.0)	1 (100.0)
Swelling	0 (0.0)	0 (0.0)	14 (100)
None	0 (0.0)	1 (7.1)	13 (92.9)

Abbreviation: AE, adverse event.

### 3.4 Tolerability/Flexibility of the treatment

The feasibility of HArmonyCa™ treatment in the presence of different factors was investigated. At baseline, 27 patients were affected by a systemic disease and received medication for it (Table 1). Moreover, in the 6 months before or after HArmonyCa™ treatment, several patients underwent cosmetic procedures (86 prior and 76 after). These cosmetic procedures included injections of botulinum toxin or other dermal fillers, laser treatment, and surgery (Tables 1 and 2). No incompatibilities or AEs were reported in patients under medication for systemic diseases or who underwent cosmetic procedures before or after HArmonyCa™ injection. Additionally, at the 9‐month follow‐up visit, several patients reported weight changes (Table 2); however, weight gain or loss did not influence the effectiveness and safety of HArmonyCa™ treatment. Finally, as the study occurred during the anti‐COVID‐19 vaccination campaign, patients received vaccinations close to the HArmonyCa™ treatment. As for other medications, no related AEs were registered. This study indicates that HArmonyCa™ treatment is safe and effective also in combination with other medications and aesthetic procedures.

In conclusion, this study shows that a single HArmonyCa™ treatment for facial rejuvenation in patients with concomitant skin conditions is highly effective and satisfactory, as assessed by physicians and patients. Only minor AEs were registered, which resolved spontaneously. Moreover, in this study, HArmonyCa™ treatment was fully compatible with other medications and cosmetic procedures.

## 4 DISCUSSION

As the appearance of the face is a key element in social interactions, facial aging, and dermal conditions may lead to discomfort and alter our sense of beauty.^7^ Facial aging is a multifactorial process, with UV‐related photoaging being one of the major causes.^19^ Aging processes lead to changes in facial morphology, resulting in loss of volume, skin laxity, and disruption of HA, collagen, and elastic fibers' networks.^20^ In addition, facial skin can be affected by several dermal conditions. Rosacea, acne, and resulting scarring are among the most widespread and significantly impact facial appearance.^6^ Injectable dermal fillers are a popular approach to achieve facial rejuvenation and improve skin quality, as they offer a safe, minimally invasive, and effective solution.^8^ Recently, HArmonyCa™, a hybrid filler combining the actions of both HA and CaHA, has been developed.^5^

In our study, 129 patients affected by one or more facial condition(s) received one treatment with HArmonyCa™ at different facial areas. Results were evaluated by physicians after 9 months using GAIS and showed that all patients displayed a visible improvement in their face appearance and skin quality. In particular, the outcomes of most patients were assessed as “much improved,” especially for those affected by UV‐related conditions, such as photoaging and rosacea.

The high efficiency of one single treatment with HArmonyCa™ is the result of its characteristics, which combine the properties of HA and CaHA fillers.^13^ The deposition of HA in the treated areas of the face gives an immediate volumizing effect. In the meantime, deposition of CaHA microspheres forms a scaffold and stimulates the fibroblasts, which, after 1 month, start to generate new collagen, providing long‐lasting results.^14^ Combining HA and CaHA fillers is a very effective solution to compensate for the respective weaknesses and boost the respective strengths. In particular, CaHA implements neocollagenesis, while the high viscosity of HA helps to create a smoother filler that is easier to inject. HA also supports the volumizing effect, waiting for the synthesis of new collagen. This dual effect ensures an immediate effect with long‐lasting results.^11^, ^13^ In recent years, (off‐label) co‐treatments with HA and CaHA have been performed with positive results^11^, ^21^; however, the HA/CaHA combined filler HArmonyCa™ offers a ready‐to‐use product, minimizing technical errors. As HArmonyCa™ has been recently developed, only few studies are present in the literature, with most of them focusing on its employment for aesthetic facial rejuvenation.^5^, ^10^, ^14^ Only few studies investigated its effect in patients affected by skin conditions, such as actinic elastosis and facial lipoatrophy.^3^, ^15^ Interestingly, HA injections have recently been shown to be effective and safe in integrating the management of erythematous rosacea.^22^ In line with the literature, our study shows that one HArmonyCa™ treatment provides great outcomes in patients presenting facial skin conditions, such as photoaging, oily, and acne‐prone skin, rosacea, and scarring.

Our study evaluated the patients' satisfaction levels after treatment. The patients' evaluations were generally very positive and agreed significantly with the physicians' evaluations. Only one out of 129 patients perceived the result as worse than before treatment. The physician did not confirm this. The patient possibly had higher expectations for the treatment, or the immediate erythema they experienced caused the dissatisfaction. Nevertheless, this isolated case did not reflect the general outcome.

Patients' assessment of the treatments is not often investigated, and only very few studies in the literature consider the patients' evaluations.^5^, ^10^

Moreover, our study shows that HArmonyCa™ is a dynamic filler that improves skin laxity. The improvement is even more evident during facial movement. This is an essential property, as the filler resolves skin elasticity and volume loss, maintaining a natural facial appearance and mimicry. To our knowledge, this is the first study to investigate the dynamic nature of this hybrid filler, using 3D images of the face from different points of view, employing the pinch test, and during movement, such as smiling and lowering the chin. The position of the face with a lowered chin is of particular interest for two main reasons: on the one hand, it mimics the aging process of the facial tissues, on the other hand, it is a standard view of the face for people using smartphones for video calls.

According to the specific patients' needs, HArmonyCa™ was injected in different face areas using a cannula, following the retrograde linear fanning technique. This technique is commonly employed for dermal filler injections, allowing the homogeneous spread of the filler in the designated areas and achieving the most favorable results.^23^ For the purpose of this study, a consensus among physicians was established to define five entry points, which can be used to reach the desired facial areas, optimize the product's delivery, and minimize side effects.

In our study, only minor AEs were registered, which resolved spontaneously, without medical intervention, confirming the safety of the treatment and emphasizing the positive response to the product of patients affected by skin conditions. A similar conclusion was drawn in a recent study by Braz et al. that evaluated the long‐term safety data on the use of a hybrid dermal filler combining HA, CaHA, and lidocaine (L).^24^ Our study showed that HArmonyCa™ is compatible with other aesthetic treatments. Indeed, none of the patients who received different cosmetic procedures reported AEs. These data align with our previous study, where we assessed the compatibility of HArmonyCa™ treatment in combination with laser lipolysis with a 1470 nm diode laser (EndoliftX®).^14^ Notably, in this study, treatment with HArmonyCa™ demonstrated compatibility with various cosmetic procedures and diverse medications, accommodating the needs of numerous patients with different systemic illnesses. Furthermore, despite the literature reporting several AEs associated with the combination of dermal fillers and anti‐COVID‐19 vaccinations,^25^, ^26^ our study did not register any incompatibility.

This study has a few limitations, which we will discuss. First, being a real‐life retrospective study, it lacks a control population. The aim of the study was, however, not to claim non‐inferiority but to share our positive experience with the injectable. Further studies will be required to assess if this treatment is the best option for facial rejuvenation procedures in patients with concomitant skin conditions. The study's retrospective nature brings along limitations of such design, especially a selection bias. The sample population might not be representative of the target population of people who resort to facial rejuvenation procedures, leading to results that may not be generalizable.

In conclusion, this study demonstrates that one HArmonyCa™ treatment greatly improves face appearance and skin quality in patients affected by general scarring and UV‐related skin conditions. In particular, HArmonyCa™ can be defined as a dynamic filler, as it enhances skin elasticity not only in a face at rest, but mostly during facial movements. Patients were highly satisfied and in agreement with the physicians' assessments. Moreover, HArmonyCa™ treatment proved to be safe and fully compatible with different medications and other aesthetic procedures.

## AUTHOR CONTRIBUTIONS

I.P. designed the study, performed the procedures, collected the patients' information, and drafted the manuscript; A.S. performed statistical analysis; A.F. and S.M. drafted the manuscript; A.G., M.D.C., S.Q., C.S., D.B., M.P., M.C., M.T.S., N.K., and E.A. participated in the study design and provided medical expertise to define a consensus on medical procedures.

## ETHICS STATEMENTS

The authors confirm that the ethical policies of the journal, as noted on the journal's author guidelines page, have been adhered to. The study was conducted in accordance with the Declaration of Helsinki. All patients provided their informed consent.

## Data Availability

The data that support the findings of this study are available on request from the corresponding author. The data are not publicly available due to privacy or ethical restrictions.
